# Editorial: Mindfulness-based interventions in clinical settings: are there benefits for both patients and their physicians?

**DOI:** 10.3389/fpsyg.2024.1423847

**Published:** 2024-06-11

**Authors:** Petra Hanson, Rohit Shankar, Jeremy Dale, Juan V. Luciano

**Affiliations:** ^1^Warwick Medical School, University of Warwick, Coventry, United Kingdom; ^2^Warwickshire Institute for the Study of Diabetes, Endocrinology and Metabolism, University Hospitals Coventry and Warwickshire NHS Trust, Coventry, United Kingdom; ^3^University of Plymouth Peninsula School of Medicine, Truro, United Kingdom; ^4^Cornwall Partnership NHS Foundation Trust, Truro, United Kingdom; ^5^Department of Clinical and Health Psychology, Autonomous University of Barcelona, Bellaterra, Spain; ^6^Teaching, Research and Innovation Unit, Parc Sanitari Sant Joan de Déu, St. Boi de Llobregat, Spain and CIBER of Epidemiology and Public Health (CIBERESP), Madrid, Spain

**Keywords:** mindfulness, burnout - professional, wellbeing & happiness, meditation, pain

## Introduction

Humans are programmed to think about issues outside their immediate vicinity, for example past events and speculation of the future. In fact, *mind wandering* appears to be the human brain's default mode of operation (Killingsworth and Gilbert, 2010). The opposite of *mind wandering* is meditation and mindfulness.

For thousands of years, philosophical and religious traditions have studied the implications of meditation for individuals and society (Sun, 2014) concluding that happiness is found by living “in the moment”. Practitioners are trained to resist *mind wandering* and to be grounded in the “here and now”. Mindfulness is considered to be a decontextualized and “modern” form of meditation. As defined by Kabat-Zinn (1994) it is “paying attention in a particular way, on purpose, in the present moment, and nonjudgmentally.”

Different forms of mindfulness-based interventions (MBIs) and compassion-based interventions (CBIs) have been developed in recent decades with efforts to integrate Buddhist meditation to Western science. These structured protocols of MBIs and CBIs have been tested for a wide range of physical (e.g., diabetes, obesity, cardiovascular diseases, etc.) and psychiatric conditions (e.g., depression, anxiety, etc.). They have also been utilized for reducing and management of burnout and stress of healthcare professionals (Fendel et al., 2021; Goldberg et al., 2022).

The Research Topic focused on the benefits of mindfulness and compassion for patients, as well as for practitioners. The six studies are diverse. They include studies on state mindfulness psychometric measures, systematic review of cultivating self-compassion for the management of low back pain, cross sectional inquiry assessing the relationship between mindfulness state and work-life balance, a randomized control trial protocol examining the feasibility and efficacy of MBIs for both patients and professionals and a couple of pilot studies. We provide a brief outline of the included studies and offer suggestions for advancing this field.

## An outline of the studies

Burnout in health care workers is omnipresent across the world. In healthcare there are several aspects outside the individual's locus of control (admin burden, lack of staff, increase in numbers of patients). However, attempts can be made to learn to control one's internal environment and reflect on how to respond to these external factors. Innovative initiatives using mindfulness approaches strive to address this.

Server et al. study assessed the feasibility of an 8-week mindfulness program designed to reduce staff burnout working in a pain clinic in a tertiary public hospital in Barcelona, Spain. In this longitudinal cohort study daily 10-min mindfulness practice for 8 weeks was combined with virtual group sessions for 10 participants. The main outcome was feasibility of delivery of these group sessions. A high adherence (95%) and participation rate (90%) was noted. Exploratory analyses on the 10 participants showed promising results with improvement in mindfulness and burnout in physicians. This is the first study of its kind i.e., assessing mindfulness interventions in health care workers who look after people living with pain.

Roman et al. focused on improving emotional burden and work-related stressors among medical interpreters. In this mixed-methods pilot study, weekly in-person 1-h sessions adapted from Mindful Practice in Medicine were delivered for 8 weeks to 17 participants in University or Rochester Medical Centre. Results showed improved resilience, teamwork, coping, compassion satisfaction and burnout after the course and 1 month later. This was the first study of its kind to be done in medical interpreters.

Lin et al. delved into the effects of mindfulness on work-life balance, spirituality and perceived professional benefits among nurses. This cross-sectional study of 303 nurses in hospitals in Jiangxi Province, China using complex regression analysis and modeling showed a positive relationship between mindfulness traits, workplace spirituality and enhanced sense of work fulfillment.

Badia-Aguarón et al. express an intention to investigate the influence of mindfulness in 120 children with attention deficit hyperactivity disorder (ADHD) recruited at Child and Adolescent Mental Health Service of Sant Joan de Déu Terres de Lleida (Spain). The NeuroMind feasibility study protocol aims to evaluate the preliminary effectiveness of three interventions i.e., Mindfulness for Health (M4H), Cognitive Training using the NeuronUP^®^ platform (CT), and a combination of both, Mindfulness Cognitive Training (MCT). There will be 30 participants per study arm. This study, designed as a 5-month randomized controlled trial, will analyze the effectiveness of these interventions added to the standard treatment for children with ADHD. Additionally, it will explore the role of psychological variables such as emotional regulation as mediators of short- and medium-term clinical outcomes.

Meanwhile, Navarrete et al. examined the psychometric properties of two mindfulness scales in the Spanish population. The Toronto Mindfulness Scale (TMS) and the State Mindfulness Scale (SMS) were analyzed in six distinct samples from the Spanish general population. The results show a robust factor structure and adequate convergent and discriminant validity, supporting their utility in assessing state mindfulness in the Spanish population. However, it is important to highlight the poor reliability of the SMS specific factors, which suggest that the computation of sub-scale scores would not be warranted.

Greff Ballejos et al. argue that self-compassion could emerge as a promising tool to improve both pain control and mental health. Their systematic review investigates the benefits of self-compassion-related interventions in adults with low back pain. Compiled results of the four studies meeting inclusion criteria suggest that self-compassion can reduce pain intensity, psychological stress, and improve pain acceptance. However, the need for more research in this field is emphasized.

## Conclusions

This Research Topic highlights the vast potential of mindfulness-based interventions in a clinical setting and the need for further research. The interventions described have the potential to improve wellbeing and health of both clinical staff and patients. However, the small numbers of participants in the interventional studies, lack of randomization and not enough longitudinal data contribute to hesitancy of recommending these interventions at a large scale. Health care services and funding bodies should recognize mindfulness as a potential intervention and support further research.

Mindfulness has significant potential given that it can be used via self-management via user empowerment, is safe and likely to be cost effective compared to current available interventions. The only caveat is that it only works if it is practiced, but as Sharon Salzberg said “Mindfulness isn't difficult, we just need to remember to do it.”

## Author contributions

PH: Writing – original draft, Writing – review & editing. RS: Writing – review & editing. JD: Writing – review & editing. JL: Writing – original draft, Writing – review & editing.
